# Interfacial Design on Graphene–Hematite Heterostructures for Enhancing Adsorption and Diffusion towards Superior Lithium Storage

**DOI:** 10.3390/nano11010081

**Published:** 2021-01-02

**Authors:** Qian Zhang, Peide Han, Jun Mei

**Affiliations:** 1 College of Materials Science and Engineering, Taiyuan University of Technology, Taiyuan 030024, China; zhangqianlink@tyut.edu.cn; 2 School of Chemistry and Physics, Queensland University of Technology, Brisbane, QLD 4000, Australia; 3 Centre for Materials Science, Queensland University of Technology, Brisbane, QLD 4000, Australia

**Keywords:** interface, α-Fe_2_O_3_, heteroatoms, lithium storage, first principle

## Abstract

Hematite (α-Fe_2_O_3_) is a promising electrode material for cost-effective lithium-ion batteries (LIBs), and the coupling with graphene to form Gr/α-Fe_2_O_3_ heterostructures can make full use of the merits of each individual component, thus promoting the lithium storage properties. However, the influences of the termination of α-Fe_2_O_3_ on the interfacial structure and electrochemical performance have rarely studied. In this work, three typical Gr/α-Fe_2_O_3_ interfacial systems, namely, single Fe-terminated (Fe-O_3_-Fe-R), double Fe-terminated (Fe-Fe-O_3_-R), and O-terminated (O_3_-Fe-Fe-R) structures, were fully investigated through first-principle calculation. The results demonstrated that the Gr/Fe-O_3_-Fe-R system possessed good structural stability, high adsorption ability, low volume expansion, as well as a minor diffusion barrier along the interface. Meanwhile, investigations on active heteroatoms (e.g., B, N, O, S, and P) used to modify Gr were further conducted to critically analyze interfacial structure and Li storage behavior. It was demonstrated that structural stability and interfacial capability were promoted. Furthermore, N-doped Gr/Fe-O_3_-Fe-R changed the diffusion pathway and made it easy to achieve free diffusion for the Li atom and to shorten the diffusion pathway.

## 1. Introduction

Hematite (α-Fe_2_O_3_), as one of the promising electrode materials for cost-effective lithium-ion batteries (LIBs), has aroused extensive attention due to its high theoretical capacity (1415 mAh g^−1^), environmentally benign nature, and low cost without obvious safety concerns [1,2,3,4,5,6,7,8]. Similar to other metal oxide anodes, the sole utilization of pristine hematite is still challenging. α-Fe_2_O_3_ possesses a poor conductivity and will suffer obvious structural variation (>200%) during repeated lithiation/delithiation processes, resulting in undesired material pulverization, and thus a large irreversible capacity and a weak cycling stability [9,10,11,12]. To promote the lithium storage performance of the α-Fe_2_O_3_ electrode, research has demonstrated that nano-sized α-Fe_2_O_3_ possesses better electrochemical performance, which is primarily due to the shorten diffusion path for both Li ions and electrons as well as a large specific active surface in comparison with micro-sized structures [13,14,15]. However, two major drawbacks for nano-sized metal oxide particles are the nonuniform dispersion degree in the operation solutions and the easy aggregation tendency under sample drying and electrode preparation processes, which will lead to serious capacity loss and stability decay [16,17,18,19].

The coupling of nano-sized α-Fe_2_O_3_ with conductive substrates is a promising solution for enhancing ion storage performance in order to address these issues associated with low conductivity, the large structural fluctuation, and the unsatisfying dispersion level. For example, two-dimensional (2D) graphene (Gr) is known to possess a large surface area, high electrical conductivity, superior mechanical properties, excellent chemical and thermal stability, and attractive ion storage potentials [20,21,22,23]. To combine α-Fe_2_O_3_ with Gr to produce Gr/α-Fe_2_O_3_ heterostructures has been verified as an effective strategy to achieve the superior Li storage performance with high specific capacity, long cycle life, and good rate capability. Qu et al. found that the specific capacity of Gr/α-Fe_2_O_3_ heterostructured composite was as high as 930 mAh g^-1^ after 50 cycles, and this was maintained at 337 mAh g^−1^ at a high current density of 10 A g^−1^ [24]. Li et al. synthesized monolithic Gr/α-Fe_2_O_3_ heterostructure in the absence of reducing agent, and this hybrid showed good cyclability with a stable reversible capacity of 810 mAh g^−1^ at 100 mA g^−1^ after 100 cycles and a good rate performance, with the capacity of 280 mAh g^−1^ remaining at a rate of 2500 mA g^−1^ [25]. These experimental results on the Li storage properties suggest that the heterostructured interface between α-Fe_2_O_3_ and Gr may act as a crucial role for the superior performance. Unfortunately, the specific interfacial relationships for Gr/α-Fe_2_O_3_ heterostructures and the potential effects of the specific interfaces on lithium-ion storage properties have been unclear until now.

For Gr/α-Fe_2_O_3_ heterostructures, the interfacial structures are relatively complex. This is largely due to the different terminations types on the dominate (0001) surface for the corundum-type α-Fe_2_O_3_ structure [26,27,28]. There are three commonly used and chemically distinct termination types on the (0001) surface of α-Fe_2_O_3_, namely, single Fe-layer (Fe-O_3_-Fe-R), double Fe-layers (Fe-Fe-O_3_-R), and O-layer (O_3_-Fe-Fe-R) (R represents the bulk stoichiometric stacking unit) [29,30,31]. The specific relations between α-Fe_2_O_3_ structures with different termination types and the ion storage properties remain a major challenge owing to the fact that the α-Fe_2_O_3_ with a single termination type is difficult to be experimentally synthesized, and in most cases, both Fe- and O-terminated domains can be identified on the synthesized α-Fe_2_O_3_(0001) surface [28]. From this viewpoint, the in-depth theoretical understanding on the interfacial structure of Gr/α-Fe_2_O_3_ between different terminated α-Fe_2_O_3_ and Gr, and their potential effects on Li ion adsorption and diffusion behaviors are still in urgent need.

In this work, a systematical investigation was conducted on the interfacial structures and Li storage performance of different terminated Gr/α-Fe_2_O_3_ heterostructures. It is verified that the single Fe-terminated Gr/Fe-O_3_-Fe-R structure manifests a low energy diffusion barrier, small volume change, and high Li^+^ storage capacity, suggesting its practicability for high-performance Li-ion batteries in comparison with the double Fe-terminated Gr/Fe-Fe-O_3_-R and O-terminated Gr/O_3_-Fe-Fe-R systems. To further optimize the interface, we constructed some modifications on the Gr structure by introducing heterostructured active atoms (e.g., B, N, O, S, and P) into Gr skeletons. Finally, a critically analysis is given on the influences of different doped Gr structures on the interfacial stability, the active energy ion diffusion barriers, and the ion diffusion pathways. It is expected that this work can offer some new theoretical insights on the interfacial enhancement for Gr/metal oxide heterostructures, and the interfacial design principles for boosting ion storage properties for advanced rechargeable batteries.

## 2. Calculation Methodology

### 2.1. Computing Parameters

All calculations in this article were carried out by the first-principles method based on density functional theory (DFT) as implemented in the Cambridge Serial Total Energy Package (CASTEP) software. The generalized gradient approximation (GGA) with the Perdew–Burke–Ernzerhof (PBE) functional was used for the electron exchange correlation potential [32,33]. Grimme’s method was used to calculate the Van der Waals interaction between Gr and α-Fe_2_O_3_ layers [34,35]. The cut-off energy was set as 680 eV and k-point mesh was set as 4 × 4 × 1. For the geometry optimization, the lattice parameters and all atoms were allowed to be fully relaxed. The convergence tolerance in energy, the maximum displacement, and the maximum force were set at 1.0 × 10^−5^ eV/atom, 0.001 Å, and 0.03 eV/Å, respectively. Kinetic barriers were carried out using linear synchronous transit/quadratic synchronous transit (LST/QST) search with intermediate conjugate gradient refinements [36,37,38].

### 2.2. Heterostructures Construction

A 2 × 2 supercell of Gr (8 C atoms) and α-Fe_2_O_3_ (0001) (18 O atoms and 12 Fe atoms) were applied to construct Gr/α-Fe_2_O_3_ heterostructures with a 12 Å vacuum space along the z-direction to eliminate interactions between neighbor layers. The lattice constant mismatch between Gr and Fe_2_O_3_ (0001) surface was 1.2%. Monolayer pristine or heteroatoms doped Gr was placed on top of 3 different terminated α-Fe_2_O_3_(0001) surfaces. There were 3 typical theoretical models, including single Fe-terminated Gr/Fe-O_3_-Fe-R, O-terminated Gr/O_3_-Fe-Fe-R, and double Fe-terminated Gr/Fe-Fe-O_3_-R heterostructures.

The stability of Gr/α-Fe_2_O_3_ heterostructures was estimated by the binding energy (*E_b_*) as defined by Equation (1) [39]. The interfacial binding capability of Gr/α-Fe_2_O_3_ heterostructures was evaluated on the basis of the interfacial formation energy (*E_f_*) that was calculated by Equation (2) [40,41]. The adsorption energy (*E_ad_*) of Gr/α-Fe_2_O_3_ heterostructures was used to evaluated lithium adsorption behaviors as calculated by Equation (3) [42,43]. The volume expansion (*V_e_*) of Gr/α-Fe_2_O_3_ heterostructures was calculated via Equation (4) [44,45].

(1) $$E_{b}=\frac{1}{\Sigma N_{i}}\left[E_{Gr/Fe_{2}O_{3}}-\Sigma (N_{i}E_{iso}^{i})\right]$$

(2) $$E_{f}=E_{Gr/Fe_{2}O_{3}}-E_{Gr}-E_{Fe_{2}O_{3}}$$

(3) $$E_{ad}=E_{Li_{n}(Gr/Fe_{2}O_{3})}-(E_{Gr/Fe_{2}O_{3}}+n\mu_{Li})$$

(4) $$V_{e}=(V_{Li_{n}(Gr/Fe_{2}O_{3})}-V_{Gr/Fe_{2}O_{3}})/V_{Gr+}{}_{Fe_{2}O_{3}}$$

where *E*_Gr/Fe__2O__3_, *E*_Gr_, and *E*_Fe__2O__3_ represent the energies of the Gr/α-Fe_2_O_3_ heterostructures and the individual Gr and Fe_2_O_3_, respectively; *N_i_* is the total number of *i* atoms (*i* represents Fe, O, C, Li, B, N, P, or S); $E_{iso}^{i}$ is the energy of the isolated *i* atom; *V*_Li_*_n_*_(__Gr/Fe__2O__3__)_ represents the volume of *n* Li atoms adsorbed on the interface of Gr/α-Fe_2_O_3_; *V*_Gr/Fe__2O__3_ is volume of Gr/Fe_2_O_3_; and *μ*_Li_ is the chemical potential of a single isolated Li atom.

To further evaluate LIB performance in Gr/α-Fe_2_O_3_ structures, we further calculated open-circuit voltage (OCV) by Equation (5) [46,47,48].

(5) $$OCV\approx \left[E_{n_{1}}-E_{n_{2}}+(n_{2}-n_{1})\mu_{\mathrm{Li}}\right]/(n_{2}-n_{1})e$$

where *E_n_*_1_ and *E_n_*_2_ are the total energy of Gr/α-Fe_2_O_3_ heterostructure adsorbed with *n*_1_ and *n*_2_ lithium atoms, respectively.

## 3. Results and Discussion

### 3.1. Geometric Structures

**Figure 1:**
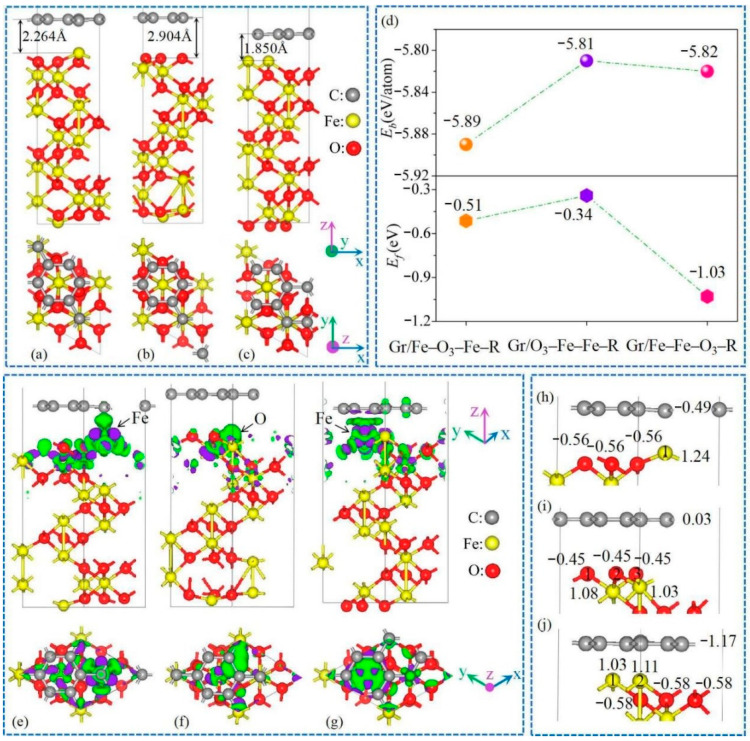
Optimized geometries of (**a**) Gr/Fe-O_3_-Fe-R, (**b**) Gr/O_3_-Fe-Fe-R, and (**c**) Gr/Fe-Fe-O_3_-R heterostructures; (**d**) binding energy (*E_b_*), interfacial formation energy (*E_f_*), and (**e**–**g**) charge difference plots of three models (isosurface level was set to 0.04 electrons/bohr^3^, green and purple areas represent charge accumulation and depletion); (**h**–**j**) transferred electrons of atoms near the interface in (**h**) Gr/Fe-O_3_-Fe-R, (**i**) Gr/O_3_-Fe-Fe-R, and (**j**) Gr/Fe-Fe-O_3_-R.

As illustrated in the optimized Gr/α-Fe_2_O_3_ heterostructures (Figure 1a–c), the vertical distances between the Gr layer and the terminated layer of α-Fe_2_O_3_ in Gr/Fe-O_3_-Fe-R, Gr/O_3_-Fe-Fe-R, and Gr/Fe-Fe-O_3_-R systems were 2.264 Å, 2.904 Å, and 1.850 Å, respectively. As illustrated in Figure 1d, *E_b_* values of Gr/Fe-O_3_-Fe-R, Gr/O_3_-Fe-Fe-R, and Gr/Fe-Fe-O_3_-R systems were all less than zero, indicating the stable structures. Furthermore, by comparison on the absolute value of the *E_f_*, we could identify that the Gr/Fe-Fe-O_3_-R structure (1.03 eV) was the most stable one, followed by the Gr/Fe-O_3_-Fe-R (0.51 eV), with the O-terminated Gr/O_3_-Fe-Fe-R system (0.34 eV) being the weakest one.

To better understand interfacial coupling effects, we studied electronic density differences for Gr/Fe-O_3_-Fe-R, Gr/O_3_-Fe-Fe-R, and Gr/Fe-Fe-O_3_-R, as shown in Figure 1e–g. Obviously, charge redistribution behaviors mainly occurred in the Gr/Fe-O_3_-Fe-R and Gr/Fe-Fe-O_3_-R systems, accompanied by the electron transfer from Fe to C atoms via the interfaces, which suggest that Fe atoms in the terminated layer donate electrons to the Gr surface. Furthermore, the charge accumulation density around C atoms in the Gr/Fe-O_3_-Fe-R system was obviously larger than that in the Gr/Fe-Fe-O_3_-R system. As for the Gr/O_3_-Fe-Fe-R system, there was almost no charge transfer through the interface, and the main force between Gr and O_3_-Fe-Fe-R was the Van der Waals interaction. In addition, Mulliken charge analysis on atoms near the interface confirmed that the Gr obtained 0.49 |e| for Gr/Fe-O_3_-Fe-R and 1.17 |e| for the Gr/Fe-Fe-O_3_-R system, and lost 0.03 |e| for the Gr/O_3_-Fe-Fe-R system in Figure 1h–j. Meanwhile, the top-level Fe atom near the interface lost 1.24 |e| in the Gr/Fe-O_3_-Fe-R system (labeled “1” in Figure 1h) and two Fe atoms in the Gr/Fe-Fe-O_3_-R system lost 1.11 |e| and 1.03 |e| (labeled “1” and “2” in Figure 1j). The interfacial atom-transferred electrons were consistent with that of the interfacial binding ability.

### 3.2. Lithium Adsorption and Diffusion Behaviours

**Figure 2:**
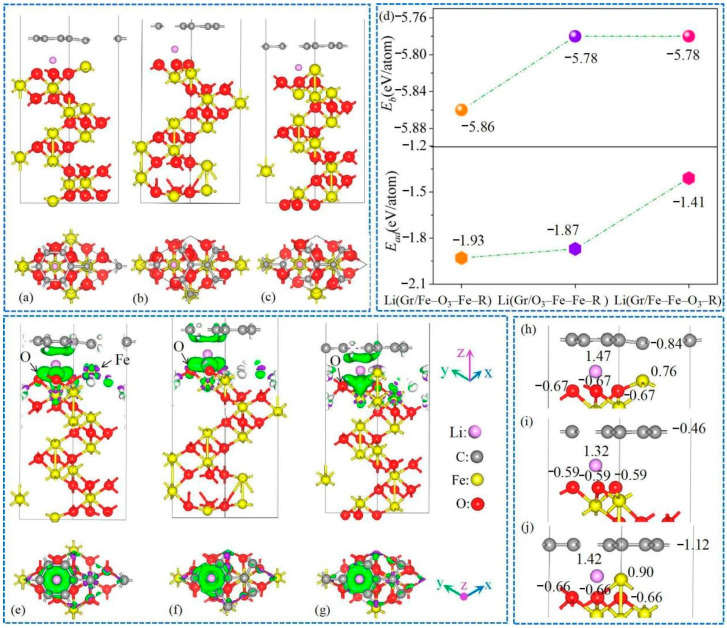
Optimized structures of (**a**) Li(Gr/Fe-O_3_-Fe-R), (**b**) Li(Gr/O_3_-Fe-Fe-R), and (**c**) Li(Gr/Fe-Fe-O_3_-R) heterostructures; (**d**) binding energy (*E_b_*), adsorption energy (*E_ad_*), and (**e**–**g**) charge difference plots of them (green and purple area represent density accumulation and depletion, isosurface level was set to 0.02 electrons/bohr^3^); (**h**–**j**) transferred electrons of atoms near the interface in (**h**) Li(Gr/Fe-O_3_-Fe-R), (**i**) Li(Gr/O_3_-Fe-Fe-R), and (**j**) Li(Gr/Fe-Fe-O_3_-R) systems.

First, to confirm the specific Li adsorption sites, we selected five Li adsorption sites (labeled as A, B, C, D, and E in Figure S1) for calculation to identify the most stable site, which were labelled as Li(Gr/Fe-O_3_-Fe-R), Li(Gr/O_3_-Fe-Fe-R), and Li(Gr/Fe-Fe-O_3_-R) systems, as demonstrated in Figure 2a–c. Compared with Gr/Fe-Fe-O_3_-R, interfacial structure was changed in Li(Gr/Fe-Fe-O_3_-R), resulting from the Fe atom labeled “1” moving down, implying initial interface structure unstable and susceptible to Li atoms. *E_b_* values in Figure 2d show that all three structures were stable. The Li adsorption ability of the Li(Gr/Fe-O_3_-Fe-R) system was the strongest, with a minimum *E_ad_* value, while the ability was the weakest of the Li(Gr/Fe-Fe-O_3_-R) system accompanied by a maximum *E_ad_* value. Charge density behavior analysis on stable Li(Gr/Fe-O_3_-Fe-R), Li(Gr/O_3_-Fe-Fe-R), and Li(Gr/Fe-Fe-O_3_-R) systems (Figure 2e–g) could verify that the most stable site for Li was in the tetrahedron gap of α-Fe_2_O_3_ terminal surface, accompanied by losing 1.47 |e|, 1.32 |e|, and 1.42 |e| in the Gr/Fe-O_3_-Fe-R, Gr/O_3_-Fe-Fe-R, and Gr/Fe-Fe-O_3_-R systems. In Figure 2h–j, the embedded Li atoms lost 1.47, 1.32, and 1.42 |e| for Li(Gr/Fe-O_3_-Fe-R), Li(Gr/O_3_-Fe-Fe-R), and Li(Gr/Fe-Fe-O_3_-R), respectively, which was consistent with the changes of adsorption ability. Owing to adsorption of Li atom, the electrons Gr lost and O atoms around the interface gained were increased in comparison with Gr/Fe-O_3_-Fe-R, Gr/O_3_-Fe-Fe-R, and Gr/Fe-Fe-O_3_-R, demonstrating that interface stability was enhanced.

**Figure 3:**
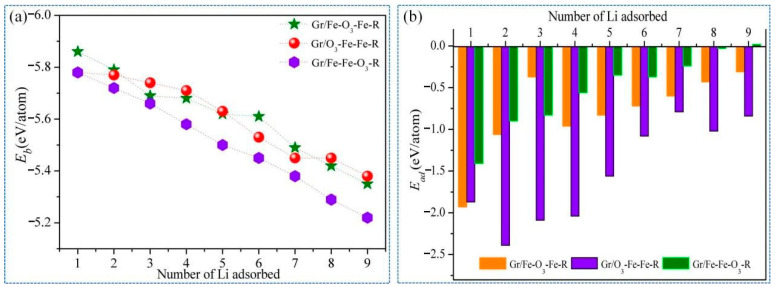
(**a**) Binding energy (*E_b_*) and (**b**) adsorption energy (*E_ad_*) of the Li*_n_*(Gr/Fe-O_3_-Fe-R), Li*_n_*(Gr/O_3_-Fe-Fe-R), and Li*_n_*(Gr/Fe-Fe-O_3_-R) systems.

*E_b_* and *E_ad_* of Li*_n_*(Gr/Fe-O_3_-Fe-R), Li*_n_*(Gr/O_3_-Fe-Fe-R), and Li*_n_*(Gr/Fe-Fe-O_3_-R) for different Li number (*n*) were calculated to evaluate structure stability and adsorption capacity, as shown in Figure 3. As the number of adsorbed Li atoms increased, the absolute values of *E_b_* and *E_ad_* decreased, resulting from the growing repulsive forces among Li atoms. It should be noted that Li atoms were unable to be stably adsorbed on the Li*_n_*(Gr/Fe-Fe-O_3_-R) system when n was equal to 9, as confirmed by a positive *E_ad_* value. Both *E_b_* and *E_ad_* absolute values for the Li*_n_*(Gr/Fe-Fe-O_3_-R) system possessed a large declining rate in comparison with the Li*_n_*(Gr/Fe-O_3_-Fe-R) and Li*_n_*(Gr/O_3_-Fe-Fe-R) systems, which showed the weakest Li adsorption capacity of the Li*_n_*(Gr/Fe-Fe-O_3_-R) system.

**Figure 4:**
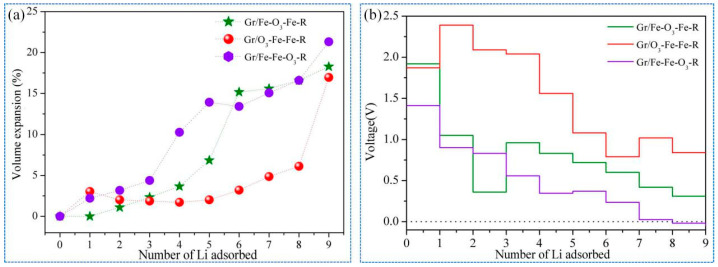
(**a**) Volume expansion (*Ve*) and (**b**) voltage figure versus number of Li for the Li*_n_*(Gr/Fe-O_3_-Fe-R), Li*_n_*(Gr/O_3_-Fe-Fe-R), and Li*_n_*(Gr/Fe-Fe-O_3_-R) configurations.

To investigate the structural variation of the Gr/α-Fe_2_O_3_ heterostructures, we calculated *Ve* during the lithiation process, as shown in Figure 4a. It was found that the volume expansion ratio of the Gr/O_3_-Fe-Fe-R system increased slowly, varying from 3.04% to 6.12% when *n* ≤ 8. However, the volume expansion of the Gr/Fe-O_3_-Fe-R system increased sharply when *n* ≥ 5, which was consistent with the Gr/Fe-Fe-O_3_-R system at *n* ≥ 4. It could be demonstrated that lithiation-induced volume expansion ratios of the Gr/Fe-O_3_-Fe-R and Gr/O_3_-Fe-Fe-R systems were less than that of the Gr/Fe-Fe-O_3_-R system, suggesting a better structural stability. Figure 4b illustrates the OCV curves along with the Li atom intercalation process. Owing to the adsorption ability, Li decreased with the increase of atomic number, and OCVs decreased as well. When *n* = 9, the OCVs of the Gr/Fe-O_3_-Fe-R and Gr/O3-Fe-Fe-R systems remained positive (0.31 and 0.84 V, respectively). However, for the Gr/Fe-Fe-O_3_-R system, the OCV became negative (−0.019 V) and the dendrite occurred [49]. On the whole, the OCV values for Gr/Fe-O_3_-Fe-R and Gr/O_3_-Fe-Fe-R systems were higher than that of the Gr/Fe-Fe-O_3_-R system. It can be inferred that Gr/Fe-O_3_-Fe-R and Gr/O_3_-Fe-Fe-R can provide high Li concentration in intercalation process while also inhibiting the undesired Li dendrite growth.

**Figure 5:**
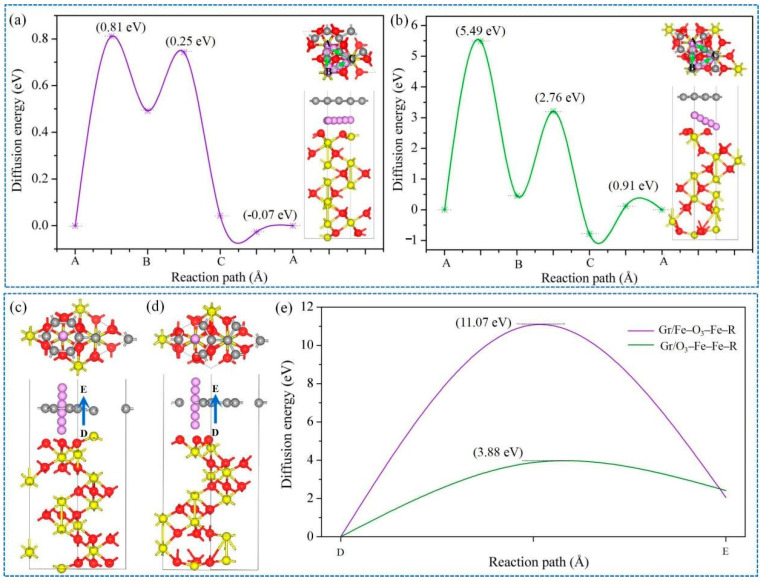
(**a**,**b**) Diffusion energy barrier profiles of A→ B, B→C, and C→A in Gr/Fe-O_3_-Fe-R and Gr/O_3_-Fe-Fe-R systems. Insets in a and b show the schematical illustration of the migration pathways; (**c**–**e**) diffusion paths and the energy barriers of D→E for Gr/Fe-O_3_-Fe-R and Gr/O_3_-Fe-Fe-R systems.

The lithium diffusion barrier is another indispensable factor for rechargeable battery. For the Gr/Fe-O_3_-Fe-R and Gr/O_3_-Fe-Fe-R systems, the optimized pathways of Li diffusion sites were site A→B→C→A, as displayed in Figure 5. The calculated results revealed that the three Li diffusion pathways of the Gr/Fe-O_3_-Fe-R system possessed much lower activation energy barriers (0.81 eV, 0.25 eV, and −0.07 eV) in comparison with these of the Gr/O_3_-Fe-Fe-R system (5.49 eV, 2.76 eV, and 0.91 eV), which implies that the Gr/Fe-O_3_-Fe-R interface could supply easy Li diffusion capability and shorten diffusion pathway. Notably, the diffusion energy barrier from C to A site was the lowest one in both Gr/Fe-O_3_-Fe-R and Gr/O_3_-Fe-Fe-R systems, demonstrating that the dominant diffusion pathway was along the interface. Furthermore, compared with the bulk Fe-O_3_-Fe-R system (Figure S2) in which the pathway from A to B site was easiest while the pathway from C to A site was the most difficult with an energy barrier of 3.07 eV, the lowest energy diffusion pathway shift from C to A in the Gr/Fe-O_3_-Fe-R system was mainly attributed to the presence of Gr. Moreover, the diffusion behaviors across the interface were evaluated, as presented in Figure 5c–e. The energy barriers of the pathway from D to E site were 11.07 and 3.88 eV for the Gr/Fe-O_3_-Fe-R and Gr/O_3_-Fe-Fe-R systems, respectively. This suggests that there were some possible vertical diffusion pathways for the Gr/O_3_-Fe-Fe-R system.

### 3.3. Interfacial Optimization by Using Heteroatoms Doped Gr

**Figure 6:**
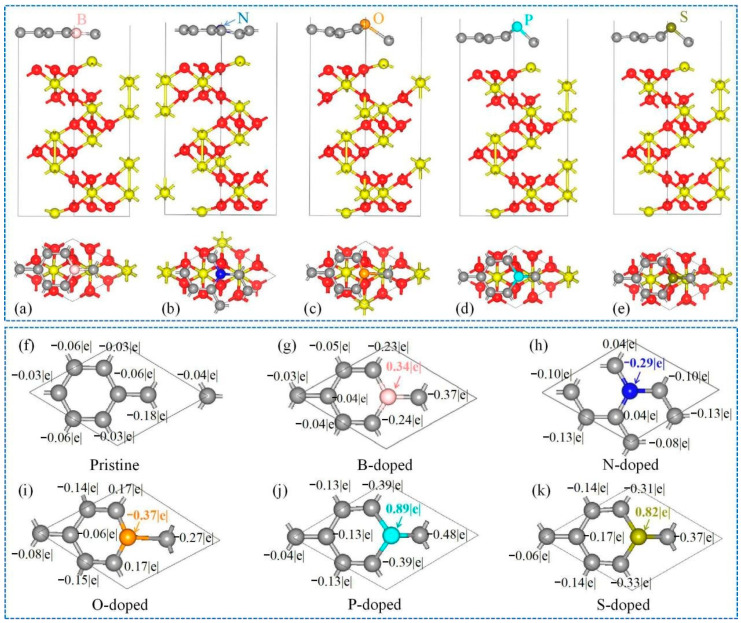
(**a**) Optimized structures for (**a**) B-, (**b**) N-, (**c**) O-, (**d**) P-, or (**e**) S-doped Gr/Fe-O_3_-Fe-R system; (**f**–**k**) transferred electrons of Gr in (**f**) Gr/Fe-O_3_-Fe-R and (**g**–**k**) M-doped Gr/Fe-O_3_-Fe-R (M = B, N, O, P, and S) systems.

**Figure 7:**
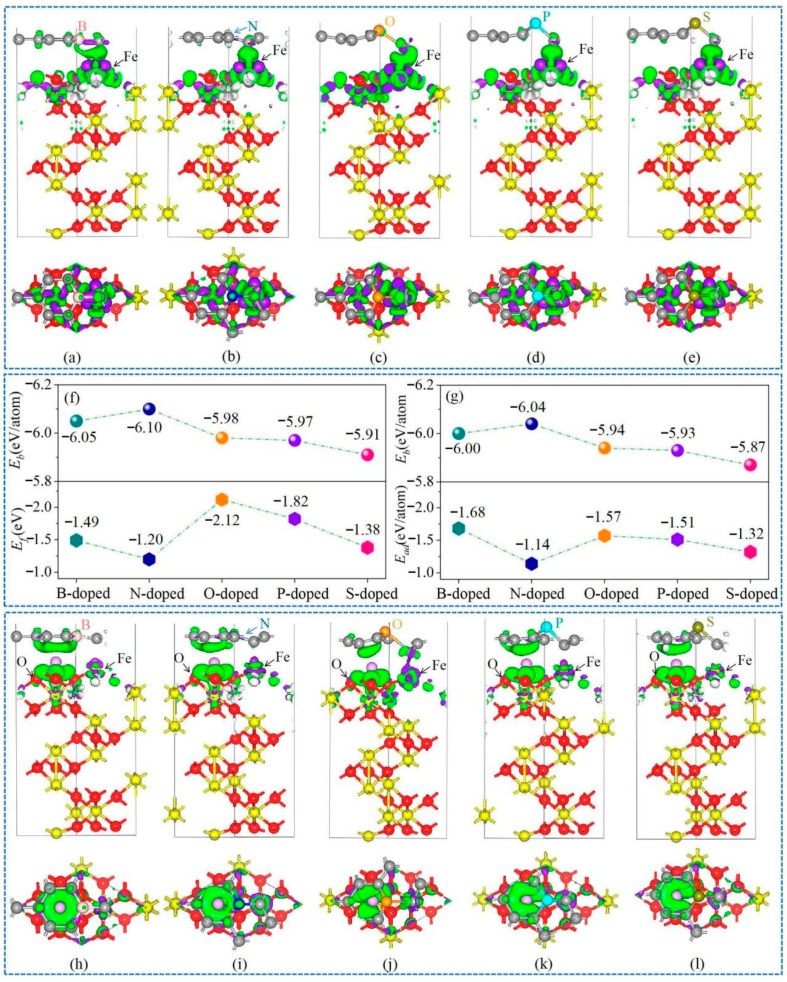
Charge difference plots for (**a**–**e**) M-doped Gr/Fe-O_3_-Fe-R systems (M = B, N, O, P, and S; isosurface level was set to 0.04 electrons/bohr^3^); (**f**) binding energy (*E_b_*) and interfacial formation energy (*E_f_*) for (**f**) M-doped Gr/Fe-O_3_-Fe-R; (**g**) binding energy (*E_b_*) and adsorption energy (*E_ad_*) for Li adsorbed on M-doped Gr/Fe-O_3_-Fe-R systems; (**h**–**l**) charge difference plots for Li adsorbed on M-doped Gr/Fe-O_3_-Fe-R systems (isosurface was set to 0.02 electrons/bohr^3^).

To further optimize the interfaces in the Gr/α-Fe_2_O_3_ heterostructures, we conducted some modifications on the Gr structure by introducing heterostructured active atoms (e.g., B, N, O, S, and P) into Gr skeleton. To simplify the calculation, we chose the Fe-O_3_-Fe-R as a typical model for α-Fe_2_O_3_. As shown in Figure 6a,b, the distance between the nearest Fe atom to the interface and B or N were 2.125 or 2.357Å, respectively, closely negative to their interface bonding performance [50]. The structures of Gr fluctuated greatly due to introducing O, P, and S, as shown in Figure 6c–e. Heteroatoms B, P, and S in the doped Gr framework lost 0.34 |e|, 0.89 |e|, and 0.82 |e|, respectively, while N and O obtained 0.29 |e| and 0.37 |e|, respectively (Figure 6f–k). Moreover, the top Fe lost 1.24 |e|, 1.29 |e|, 0.73 |e|, 0.71 |e|, 0.71 |e|, and 0.71 |e|, and meanwhile the heteroatom-doped Gr received 0.49 |e|, 0.66 |e|, 0.79 |e|, 0.73 |e|, 1.69 |e|, and 1.52 |e| in the Gr/Fe-O_3_-Fe-R, B-doped Gr/Fe-O_3_-Fe-R, N-doped Gr/Fe-O_3_-Fe-R, O-doped Gr/Fe-O_3_-Fe-R, P-doped Gr/Fe-O_3_-Fe-R, and S-doped Gr/Fe-O_3_-Fe-R systems, respectively, as shown in Figure 7a–e. Therefore, it can be inferred that the activity of Gr was promoted due to the addition of heteroatoms. Further analysis on *E_b_* and *E_f_* of the Gr/Fe-O_3_-Fe-R system with heteroatom doping was carried out (Figure 7f). The *E_b_* values of B/N/O/P/S doping on the Gr/Fe-O_3_-Fe-R systems were increased to −6.05/−6.10/−5.98/−5.97/−5.91 eV/atom, and the absolute values of *E_f_* were promoted to 1.49/1.20/2.12/1.82/1.38 eV, respectively, indicating the enhanced structural stability and the increased interface binding capability compared to the pristine Gr/Fe-O_3_-Fe-R system.

After Li was absorbed on the heteroatom-doped Gr/Fe-O_3_-Fe-R system, optimized geometries were depicted, as shown in Figure S3. *E_b_* and *E_ad_* values of Gr/Fe-O_3_-Fe-R were −5.86 and −1.93 eV/atom, respectively. If B-, N-, O-, P-, and S-doped Gr were used, the absolute values of *E_b_* increased, however, the *E_ad_* values were all reduced (Figure 7g). That means that the adsorption ability of doped Gr/Fe-O_3_-Fe-R systems for Li atom decreased in spite of the enhanced structure stability. Similar to the Gr/Fe-O_3_-Fe-R system, the electrons were transferred from Li to the O and Gr surfaces, in which Li atoms lost 1.46 |e|, 1.49 |e|, 1.38 |e|, 1.42 |e|, and 1.43 |e| in B, N, O, P, and S-doped Gr/Fe-O_3_-Fe-R systems, respectively, as shown in Figure 7h–l.

**Figure 8:**
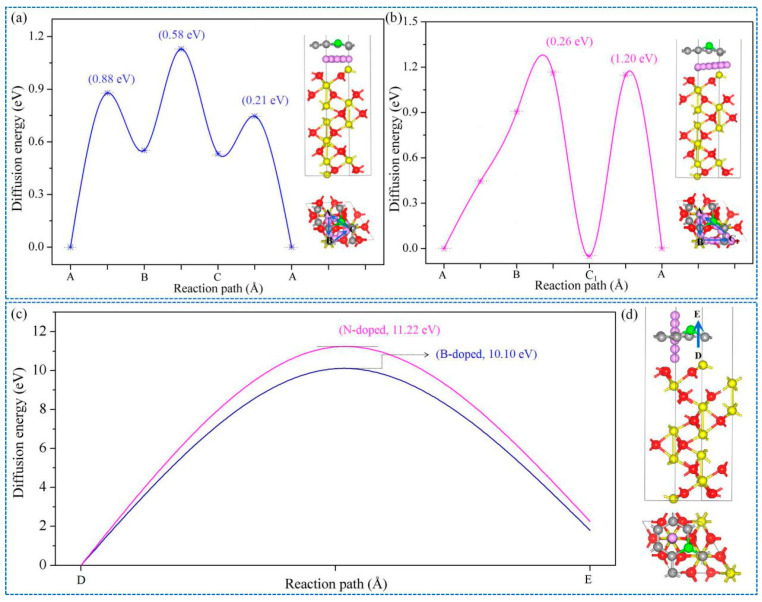
Energy barrier profiles for Li atom from A to B, B to C (C1), and C (C1) to A in (**a**) B- and (**b**) N-doped Gr/Fe-O_3_-Fe-R system; (**c**,**d**) energy barriers and diffusion paths for Li atom from D to E in B and N-doped Gr/Fe-O_3_-Fe-R.

It is well known that B and N can be easily incorporated into Gr due to them having roughly the same atomic radius as C [51]. Moreover, N and B are typical n-type and p-type donors with different effects on electronic structures of Gr [52]. In addition, heteroatoms B and N modifying Gr contribute to better structural stability of Gr/Fe-O_3_-Fe-R in comparison with introducing O, P, and S, as shown in Figure 7f–g. Consequently, the energy barrier profiles for Li atom of the three pathways (A→B, B→C (C1), and C (C1) →A site) in the B- and N-doped Gr/Fe-O_3_-Fe-R systems were conducted to evaluate the intercalation/deintercalation process. As demonstrated in Figure 8a–b, B-doped Gr exhibited little effect on the diffusion barrier from A to B site, whereas energy barriers of B→C and C→A were increased. N doping Gr changed the location of site C to C1, as shown in the illustration in Figure 8b. Moreover, embedding of N was able to alter the easiest pathway from C→A to A→B. Meanwhile, Li atom was able to diffuse freely from site A to B. As for the vertical diffusion pathway (D→E), as calculated in Figure 8c–d, the presence of B could reduce the energy barriers to 10.10 eV. On the contrary, the implantation of N could further increase the diffusion barrier.

To summarize, the presence of heteroatom-doped Gr in the Gr/α-Fe_2_O_3_ heterostructures could enhance the structural stability and interfacial bonding capability. *E_b_* absolute values were promoted from 5.89 eV to 6.05, 6.10, 5.98, 5.97, and 5.91 eV for B-, N-, O-, P-, and S-doped Gr/Fe-O_3_-Fe-R, respectively. *E_f_* absolute values were also clearly increased from 0.51 eV to 1.49, 1.20, 2.12, 1.82, and 1.38 eV, respectively. N-doping shortened the diffusion pathway and made free diffusion become possible, compared with heteroatom B.

## 4. Conclusions

On the basis of first-principle calculations, we conducted a systematical investigation on the interfacial structures, interface bonding capability, intercalation process, and Li diffusion behavior of three terminated Gr/α-Fe_2_O_3_ heterostructures as well as Li storage performance. These results show that the Gr/Fe-O_3_-Fe-R system possesses good structural stability, high adsorption ability, small volume expansion, low energy barriers, and a short diffusion pathway. To further optimize the interface, we conducted some modifications on the Gr structure by introducing heterostructured active atoms (e.g., B, N, O, S, and P) into Gr skeletons. Through a critical analysis on the influences of different heteroatom-doped Gr, we can conclude that structural and interfacial stability were promoted. Moreover, it was easier for the Li atom to migrate along the interface, and the presence of N-doped Gr possessed a free diffusion pathway. It is hoped that the present work paves the way for understanding interface properties and achieving Li rapid diffusion with a low barrier of Gr/transition metal oxides through tuning the interface microstructure.

## Data Availability

The data presented in this study are available on request from the corresponding author.

## Supplementary Materials

The following are available online at https://www.mdpi.com/2079-4991/11/1/81/s1: Figure S1: Adsorption sites A, B, C, D, and E for Li atom in (**a**,**d**) Gr/Fe-O3-Fe-R, (**b**,**e**) Gr/O3-Fe-Fe-R, and (**c**,**f**) Gr/Fe-Fe-O3-R systems. Figure S2: Energy barrier profiles for Li atom in α-Fe_2_O_3_ (0001) surface. Figure S3: Optimized structures of Li adsorbed in (**a**–**j**) M-doped Gr/Fe-O3-Fe-R systems (M = B, N, O, P, and S).
