# Supplementary material for: Interfacial Design on Graphene–Hematite Heterostructures for Enhancing Adsorption and Diffusion towards Superior Lithium Storage
Source: Nanomaterials (Basel). 2021 Jan 2;11(1):81. doi: 10.3390/nano11010081 (PMC7823445; doi:10.3390/nano11010081)
Supplement: Supplementary file 1 [file nanomaterials-11-00081-s001.pdf]

Supplementary Materials

Interfacial Design on Graphene–Hematite Heterostructures for Enhancing Adsorption and Diffusion towards Superior Lithium Storage

Qian Zhang [1], Peide Han [1,*] and Jun Mei [2,3,*]

[1] College of Materials Science and Engineering, Taiyuan University of Technology, Taiyuan 030024, China; zhangqianlink@tyut.edu.cn
[2] School of Chemistry and Physics, Queensland University of Technology, Brisbane, QLD 4000, Australia
[3] Centre for Materials Science, Queensland University of Technology, Brisbane, QLD 4000, Australia
* Correspondence: hanpeide@tyut.edu.cn (P.H.); j2.mei@qut.edu.au (J.M.)

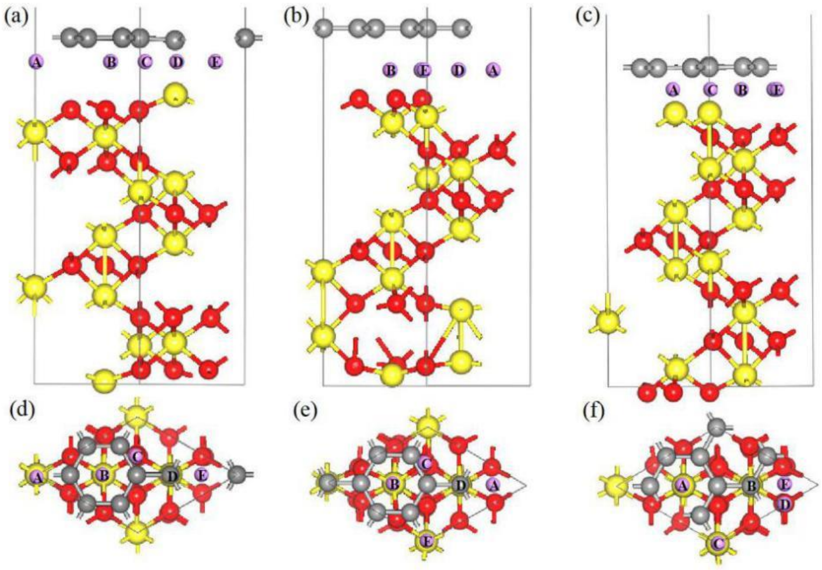

Figure S1. Adsorption sites A, B, C, D and E for Li atom in (**a,d**) Gr/Fe-O_3-Fe-R, (**b,e**) Gr/O_3-Fe-Fe-R and (**c,f**) Gr/Fe-Fe-O_3-R systems.

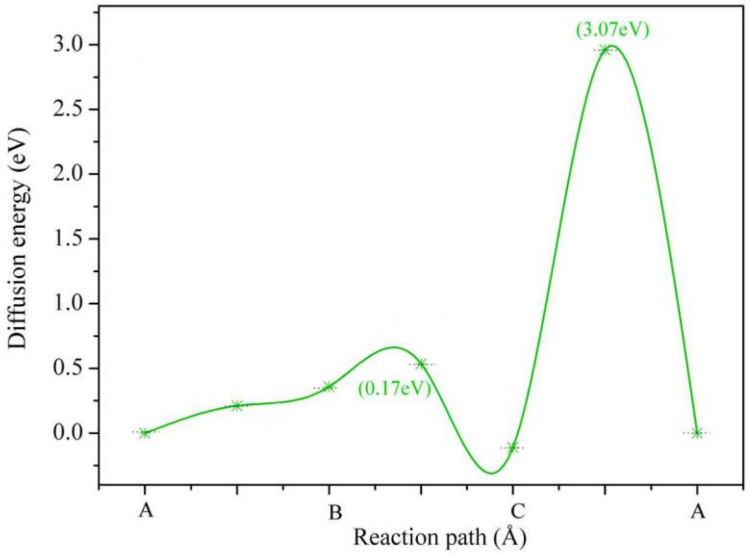

Figure S2. Energy barrier profiles for Li atom in α-Fe_2O_3 (0001) **s**urface.

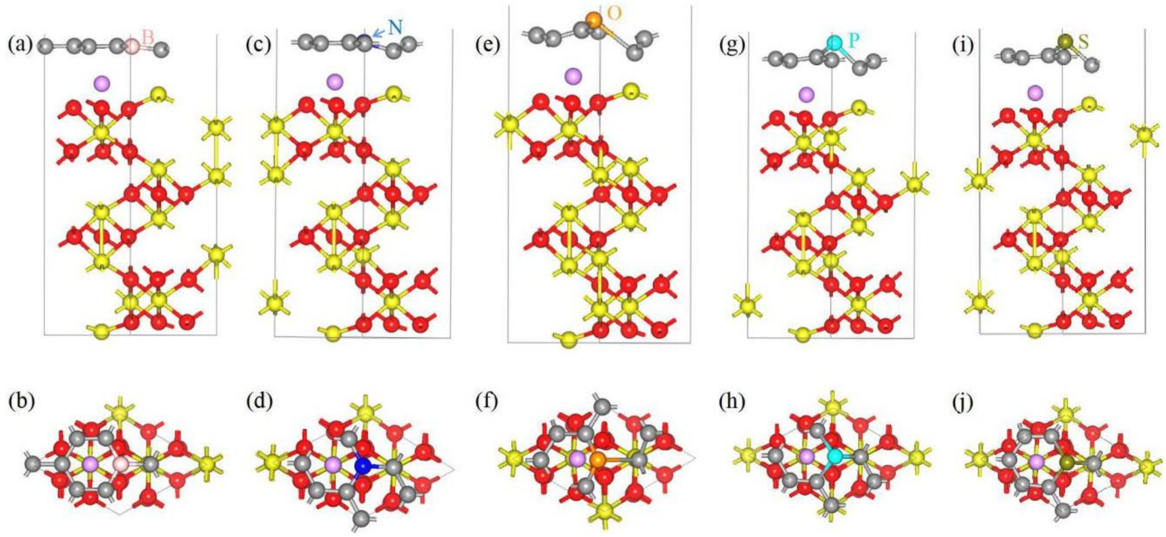

Figure S3. Optimized structures of Li adsorbed in (**a–j**) M-doped Gr/Fe-O_3-Fe-R systems (M = B, N, O, P and S).
